# Hormonal and inflammatory responses to high‐intensity functional training in male soldiers

**DOI:** 10.14814/phy2.70592

**Published:** 2025-10-02

**Authors:** Joonas Helén, Heikki Kyröläinen, Tommi Ojanen, Kai Pihlainen, Risto Heikkinen, Jani P. Vaara

**Affiliations:** ^1^ Department of Leadership and Military Pedagogy National Defence University Helsinki Finland; ^2^ Faculty of Sport and Health Sciences University of Jyväskylä Jyväskylä Finland; ^3^ Human Performance Division Finnish Defence Research Agency Riihimäki Finland; ^4^ Training Division Defence Command, Finnish Defence Forces Helsinki Finland; ^5^ Statistical Analysis Services Analyysitoimisto Statisti Oy Jyväskylä Finland

**Keywords:** biomarkers, fitness, high intensity, hormones, inflammation, military

## Abstract

This study investigated the effects of high‐intensity functional training (HIFT) on hormonal and inflammatory biomarker responses during military service. One hundred and twenty‐seven male conscripts were assessed over a 19‐week training period. The experimental group (EXP: *n* = 64) followed a structured HIFT program, while the control group (CON: *n* = 63) adhered to conventional military physical training. Blood samples were collected at baseline (PRE), week 10 (MID), and post‐intervention (POST) to measure serum testosterone, cortisol, testosterone/cortisol ratio, insulin‐like growth factor 1 (IGF‐1), sex hormone‐binding globulin (SHBG), high‐sensitivity C‐reactive protein (hs‐CRP), interleukin 6 (IL‐6), and tumor necrosis factor alpha (TNF‐α). Body composition and physical performance were also measured. Serum testosterone increased in both groups between PRE and POST (EXP: +3.3 ± 3.8 nmol·L^−1^, *p* < 0.001; CON: +3.7 ± 3.4 nmol·L^−1^, *p* < 0.001), while cortisol remained unchanged. Testosterone/cortisol ratio increased in both groups (+0.010 ± 0.010, *p* < 0.001 for both). IGF‐1 increased in CON (+4.4 ± 5.9 nmol·L^−1^, *p* < 0.001) and SHBG increased in EXP (+3.1 ± 9.0 nmol·L^−1^, *p* = 0.005). Inflammatory biomarkers (hs‐CRP, IL‐6) decreased in both groups. No adverse biomarker responses were observed, suggesting that HIFT was well tolerated during military service.

## 1 INTRODUCTION

Physical fitness is crucial for soldiers due to the demanding nature of their duties, which often involve exposure to multiple stressors, such as high levels of physical exertion, sleep deprivation, energy deficiency, and psychological stress. These stressors impose significant challenges on soldier physiology, potentially leading to adverse effects on physical performance and health. Therefore, when implementing physical training programs for soldiers, careful consideration of the total training load is required (Kyröläinen et al., 2018). Existing literature provides a relatively strong foundation on hormonal and inflammatory responses measured in blood during military training, with conflicting results that are heavily influenced by differences in demands and duration of training (Beckner et al., 2022; Granlund et al., 2023). Observations from these studies can be utilized as part of assessing the total physiological strain to prevent excess fatigue or overreaching. However, there is still a lack of comprehensive longitudinal data on the physiological responses, as indicated by various blood biomarkers, to different types of physical training programs in a military context. A deeper understanding of the overall stress is necessary for the endorsement of different training approaches.

Circulating concentrations of testosterone, cortisol, and insulin‐like growth factor 1 (IGF‐1) are commonly used to assess stress responses in soldiers during military training (Nindl et al., 2007; Tanskanen et al., 2011). Although resting levels of these hormones may not serve as fully valid biomarkers to monitor physiological strain or diagnose overtraining syndrome (Cadegiani & Kater, 2017), they offer an insight into anabolic and catabolic processes in the body, reflecting the overall physiological readiness (Beckner et al., 2022). In particular, the testosterone/cortisol ratio has been identified as a useful biomarker for monitoring responses to training stress in soldiers (Tait et al., 2024; Tanskanen et al., 2011). Sex hormone‐binding globulin (SHBG), a protein that binds circulating sex hormones, is also often measured in military studies in an attempt to assess the levels of bioavailable testosterone and thus, anabolic potential of soldiers (Henning et al., 2014). Tanskanen et al. (2011) reported that soldiers who were classified as overreached after 12 weeks of basic military training had higher concentrations of SHBG throughout the training period than soldiers who were not classified as overreached.

In addition to the hormonal profile, inflammatory biomarkers are often measured to monitor both physiological strain and recovery in soldiers. Alterations in acute‐phase proteins, such as C‐reactive protein (CRP), and pro‐inflammatory cytokines, such as interleukin 6 (IL‐6) and tumor necrosis factor alpha (TNF‐α), are influenced by the type and intensity of military training. Levels of these biomarkers typically increase during the most physically demanding training periods, reflecting an acute inflammatory response to physical stress (Beckner et al., 2022; Granlund et al., 2023).

Since military training often involves high volumes of low‐intensity physical activity, which has been shown to negatively affect the optimal development of soldiers' physical performance, adoption of high‐intensity training methods may be recommended (Kyröläinen et al., 2018). The present study is part of the same intervention that we previously reported (Helén et al., 2023), where the primary focus was on changes in physical performance and body composition. In contrast, the current manuscript investigates hormonal and inflammatory biomarker responses to the same training program and their associations with physical fitness outcomes, thereby extending the earlier findings and conclusions. To our knowledge, only Drain et al. (2017) have compared the effects of a high‐intensity, low‐volume training with traditional low‐intensity, high‐volume training on serum hormones during military training. While both groups in their study showed some unfavorable changes in hormonal profiles, the stress response seemed to be attenuated in the high‐intensity training group. To enhance the understanding of this matter, the purpose of the present study was to assess the effects of high‐intensity functional training (HIFT) on hormonal and inflammatory biomarker responses during military training.

## 2 METHODS

### 2.1 Participants

This study was part of the same intervention previously reported in Helén et al. (2023), which examined changes in physical performance and body composition. Conscripts who had enrolled in compulsory military service were recruited for the present study. All participants underwent a medical screening at the beginning of their military service, which confirmed that they were healthy and capable of participating in both the military training and the present study. No additional exclusion criteria were applied. Two companies that began their service at the same time and at the same garrison were designated as the experimental (EXP) and control (CON) groups. Of the 243 male conscripts who volunteered to participate, 127 individuals (EXP: *n* = 64, age 19 ± 1 years [range 18–24 years], body mass 73.6 ± 12.5 kg, height 178 ± 7 cm; CON: *n* = 63, age 19 ± 1 years [range 18–25 years], body mass 73.3 ± 11.7 kg, height 179 ± 6 cm) completed the study. The reduction in participants was primarily due to transfers to other units and secondarily due to cessation of military service. Six participants (EXP 2; CON 4) dropped out due to a lack of motivation to participate. No women were included in the study, as the selected units consisted exclusively of men. At baseline, 29% of the participants reported engaging in vigorous exercise at least three times per week, 15% reported no regular exercise, 19% were current smokers and regarding alcohol use, 3% consumed alcohol more than three times per week while 11% reported complete abstinence. The participants provided written informed consent after being informed about the study design and potential risks. The study was conducted in accordance with the Declaration of Helsinki and received approval from the Ethical Committee of the Central Finland Health Care District (HUS‐1557‐2018‐8) and the Finnish Defence Forces (AP10027).

### 2.2 Procedures

Blood sample collection and measurements of body composition and physical fitness were conducted at three time points: 1 week prior to the start of the 19‐week physical training intervention (PRE), at training week 10 (MID), and 1 week after the intervention (POST). Body composition (body mass, muscle mass, and body fat mass) was measured in the morning after an overnight fast with segmental multifrequency bioimpedance analysis (InBody 720/770; Biospace Co. Ltd., Seoul, South Korea) according to the manufacturer's instructions. Immediately following the body composition assessment, blood samples were drawn from the antecubital vein. Participants were instructed to refrain from vigorous physical activity for 24 h prior to sampling. Samples were centrifuged at 2000×*g* for 10 min (Megafuge 1.0 R, Heraeus, Germany), aliquoted, frozen, and transported to the laboratory for analysis. Serum concentrations of testosterone, cortisol, IGF‐1, SHBG, high‐sensitivity C‐reactive protein (hs‐CRP), and IL‐6 were analyzed by an immunoassay system (Siemens IMMULITE 2000 XPi, Siemens Healthineers, Erlangen, Germany). The sensitivity and inter‐assay coefficient of variation were 0.5 nmol·L^−1^ and 7.8% for testosterone, 5.5 nmol·L^−1^ and 6.5% for cortisol, 2.6 nmol·L^−1^ and 7.8% for IGF‐1, 0.02 nmol·L^−1^ and 5.7% for SHBG, 0.2 mg·L^−1^ and 7.3% for hs‐CRP, and 2 pg·mL^−1^ and 7.2% for IL‐6. TNF‐α was analyzed using an ELISA kit (R&D Systems, Minneapolis, MN, USA) and DYNEX DS2® (DYNEX® Technologies, Chantilly, VA, USA). The sensitivity and inter‐assay coefficient of variation were 0.1 pg·mL^−1^ and 6.7% for TNF‐α.

Physical fitness measurements included the 12‐min running test (total distance covered) to assess aerobic capacity, together with six muscular strength and power tests. Maximal isometric force of the upper and lower body was assessed in a seated position using an electromechanical leg and bench press dynamometer. In the bench press, the elbow angle was fixed at 90° and the bar was set at shoulder height, while in the leg press, the knee angle was adjusted to 107°. Participants performed two maximal attempts in both tests, separated by at least 30 s of rest, and peak force (N) was recorded. Upper body power was measured with a seated medicine ball throw. Participants sat on the floor with legs extended, keeping the back firmly against the wall. From this position, they were instructed to throw a 2‐kg ball forward from the chest with both hands while maintaining contact with the wall. The distance was measured from the wall to the midpoint of the landing spot using a tape measure fixed securely to the floor. Lower body peak power was assessed with a standing long jump, performed with a countermovement and arm swing. Muscular endurance of the abdominal and hip‐flexor muscles was assessed with 1‐min sit‐ups and upper body muscular endurance with 1‐min push‐ups. The total number of repetitions was recorded in both tests. Results of the six strength tests were converted into Z‐scores and then summed to generate a composite muscle fitness score.

The training intervention has been described in detail previously (Helén et al., 2023). Briefly, participants in the EXP group trained with concurrent strength and endurance exercises emphasizing high‐intensity functional training (HIFT). Sessions (60 min) included a standardized warm‐up, one to two strength exercises performed to volitional fatigue, and a HIFT workout with circuit‐style training using bodyweight, sandbags, and kettlebells. Training intensity and volume were progressively increased, and all sessions were supervised by drill instructors. The CON group trained according to the standard physical training guidelines of the Finnish Defence Forces, primarily involving running, ball games, and calisthenics, and small amounts of swimming, orienteering, self‐defense, and resistance training at the gym. Physical activity outside of the intervention was not controlled; participants followed their normal military training schedules and could engage in voluntary activity during leisure time. During the 19‐week intervention, the total training volume was 46 and 42 h for EXP and CON, respectively, with 30 h dedicated to HIFT in EXP. Training volume was not evenly distributed across phases: in EXP, 27 h (59%) occurred during PRE–MID and 19 h (41%) during MID–POST, while in CON, 26 h (62%) occurred during PRE–MID and 16 h (38%) during MID–POST.

### 2.3 Statistical analyses

Changes within and between groups throughout the study period were estimated using a linear mixed‐effects model. Inclusion in the statistical analysis required that each participant had data from at least two of the three measurement time points. The linear mixed‐effects model allowed all available observations to be included in the analyses despite occasional missing values in the outcome variables. Logarithmic transformations were applied to skewed outcome variables. The normality of the residuals was tested, and the homogeneity of variance in the residuals was visually diagnosed. For outcome variables with problematic residuals, the results were also verified by nonparametric Mann–Whitney *U*‐tests. Interactions between groups and time were examined using the *F*‐test (Satterthwaite's method). Tukey's post hoc test was employed for within‐group pairwise comparisons across different time points. Pearson's correlation coefficients were calculated to assess relationships between absolute changes in measured variables. Statistical analysis was performed using R (version 4.4.1). All data are reported as mean ± standard deviation (SD). The significance level was set at *p* < 0.05.

## 3 RESULTS

**FIGURE 1:**
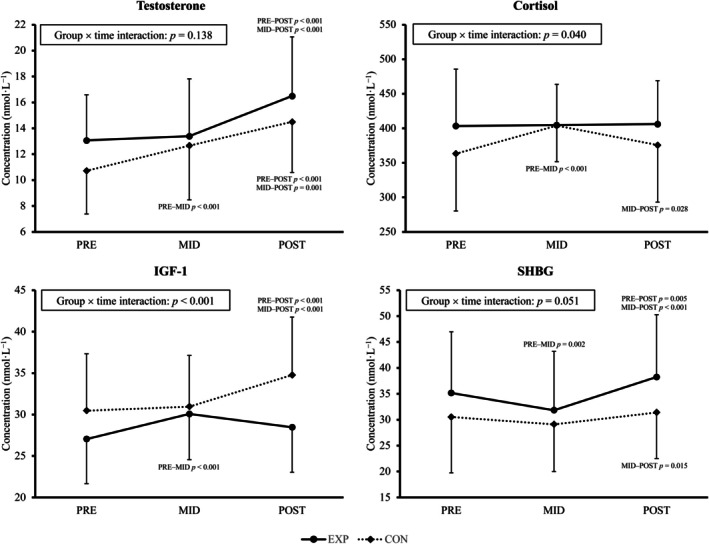
Mean (±SD) changes in serum testosterone, cortisol, insulin‐like growth factor 1 (IGF‐1), and sex hormone‐binding globulin (SHBG) in the experimental (EXP) and control (CON) training groups.

Figure 1 shows the changes in testosterone, cortisol, IGF‐1, and SHBG across the study period in EXP and CON. A significant group × time interaction was observed for serum cortisol and IGF‐1. In addition, a borderline significance was observed for SHBG. Serum testosterone increased in both groups between PRE and POST (EXP: +3.3 ± 3.8 nmol·L^−1^ [+28 ± 29%], *p* < 0.001; CON: +3.7 ± 3.4 nmol·L^−1^ [+44 ± 48%], *p* < 0.001). Cortisol increased in CON between PRE and MID (+43 ± 88 nmol·L^−1^ [+17 ± 30%], *p* < 0.001), but returned to baseline level in POST, whereas EXP showed no change across time points. Testosterone/cortisol ratio increased in both groups (EXP: PRE 0.034 ± 0.011, POST 0.042 ± 0.013, change +0.010 ± 0.010 [+28 ± 38%], *p* < 0.001; CON: PRE 0.031 ± 0.012, POST 0.040 ± 0.012, change +0.010 ± 0.010 [+44 ± 60%], *p* < 0.001). IGF‐1 increased in CON (+4.4 ± 5.9 nmol·L^−1^ [+17 ± 22%], *p* < 0.001) between PRE and POST, while no change was observed in EXP. In contrast, SHBG increased in EXP (+3.1 ± 9.0 nmol·L^−1^ [+12 ± 29%], *p* = 0.005) between PRE and POST, while no change was observed in CON.

Among inflammatory biomarkers (Table 1), similar decreases in hs‐CRP (EXP: −1.5 ± 2.7 mg·L^−1^ [−19 ± 142%], *p* < 0.001; CON: −1.8 ± 2.5 mg·L^−1^ [−34 ± 65%], *p* < 0.001) and IL‐6 (EXP: −1.3 ± 3.7 pg·mL^−1^, *p* = 0.005; CON: −1.4 ± 4.7 pg·mL^−1^, *p* = 0.015) were observed in both groups between PRE and POST. TNF‐α showed a minor increase in EXP (+0.2 ± 0.2 pg·mL^−1^ [+24 ± 30%], *p* < 0.001), but remained unaltered in CON.

**TABLE 1:** Mean (±SD) changes in inflammatory biomarkers in the experimental (EXP) and control (CON) training groups.

	PRE	MID	POST	Group × time interaction
hs‐CRP (mg·L^−1^)				
EXP	2.2 ± 2.3	1.6 ± 1.9	0.8 ± 1.2^*,‡^	*p* = 0.687
CON	2.4 ± 2.5	1.8 ± 1.7	1.2 ± 1.7^*,‡^	
IL‐6 (pg·mL^−1^)				
EXP	2.8 ± 3.7	1.9 ± 1.6	1.2 ± 2.2*	*p* = 0.975
CON	3.4 ± 4.6	2.6 ± 3.0	2.0 ± 1.3*	
TNF‐α (pg·mL^−1^)				
EXP	0.8 ± 0.2	1.0 ± 0.3^†^	1.0 ± 0.2*	*p* < 0.001
CON	0.8 ± 0.3	0.9 ± 0.2^†^	0.8 ± 0.2^‡^	

*Note*: Significant (*p* < 0.05) change: *: pre‐post, ^†^: pre‐mid, ^‡^: mid‐post.

Abbreviations: hs‐CRP, high‐sensitivity C‐reactive protein; IL‐6, interleukin 6; TNF‐α, tumor necrosis factor alpha.

Changes in body composition, 12‐min run, and muscle fitness score are presented in Table 2. A significant group × time interaction occurred for body mass, muscle mass, and muscle fitness score. Body mass decreased (−1.4 ± 4.4 kg, *p* = 0.004) between PRE and POST in EXP, which was mostly due to a reduction (−1.0 ± 3.6 kg, *p* = 0.008) in body fat mass. An increase (+0.7 ± 1.6 kg, *p* < 0.001) in muscle mass was observed in CON, while body mass remained unaltered. The performance in the 12‐min run improved in both groups, with EXP demonstrating a notably greater increase (+250 ± 295 m [+12 ± 14%], *p* < 0.001) compared to CON (+104 ± 278 m, [+6 ± 14%], *p* = 0.005). Muscle fitness score increased in EXP (+0.11 ± 0.33, *p* = 0.027), but remained unaltered in CON between PRE and POST.

**TABLE 2:** Mean (±SD) changes in body composition, 12‐min run, and muscle fitness score in the experimental (EXP) and control (CON) training groups.

	PRE	MID	POST	Group × time interaction
Body mass (kg)				
EXP	73.6 ± 12.5	73.4 ± 12.1	72.4 ± 10.6*	*p* = 0.003
CON	73.3 ± 11.7	73.4 ± 10.1	74.3 ± 9.5	
Muscle mass (kg)				
EXP	34.9 ± 4.9	35.0 ± 5.2	34.7 ± 4.6	*p* < 0.001
CON	34.5 ± 4.1	34.8 ± 3.9^†^	35.2 ± 3.9*	
Body fat mass (kg)				
EXP	12.2 ± 7.1	11.9 ± 5.9	11.3 ± 5.1*	*p* = 0.369
CON	12.2 ± 7.1	11.9 ± 6.2	12.2 ± 5.7	
12‐min run (m)				
EXP	2246 ± 287	2437 ± 285^†^	2491 ± 327*	*p* = 0.024
CON	2206 ± 324	2300 ± 323^†^	2316 ± 289*	
Muscle fitness score				
EXP	0.00 ± 0.79	0.20 ± 0.75^†^	0.09 ± 0.71*	*p* < 0.001
CON	−0.09 ± 0.67	−0.07 ± 0.67	−0.19 ± 0.69	

*Note*: Significant (p < 0.05) change: *: pre‐post, †: pre‐mid.

In both groups, the change in SHBG between PRE and POST correlated with the change in body mass (EXP: *r* = −0.27, *p* = 0.034; CON: *r* = −0.56, *p* < 0.001) and muscle mass (EXP: *r* = −0.36, *p* = 0.005; CON: *r* = −0.50, *p* < 0.001) and with body fat mass in CON (*r* = −0.35, *p* = 0.014). The change in IGF‐1 correlated with the change in body mass (*r* = −0.26, *p* = 0.043) and body fat mass (*r* = −0.28, *p* = 0.031) in EXP. In both groups, the change in hs‐CRP correlated with the change in body mass (EXP: *r* = 0.28, *p* = 0.036; CON: *r* = 0.38, *p* = 0.018) and body fat mass (EXP: *r* = 0.36, *p* = 0.006; CON: *r* = 0.53, *p* = 0.001). In CON, the change in IL‐6 correlated with the change in muscle mass (*r* = −0.36, *p* = 0.010). The change in muscle fitness score correlated with the change in SHBG (*r* = −0.35, *p* = 0.010) in EXP, and with the change in testosterone (*r* = −0.34, *p* = 0.036) in CON. The change in 12‐min run correlated with the change in testosterone/cortisol ratio (*r* = −0.36, *p* = 0.009) and IGF‐1 (*r* = 0.33, *p* = 0.016) in EXP, and with the change in SHBG (*r* = 0.32, *p* = 0.048) and hs‐CRP (*r* = −0.58, *p* < 0.001) in CON.

## 4 DISCUSSION

The primary findings of the present study were that, while significant changes occurred in serum hormones and inflammatory biomarkers during military service, these changes were predominantly favorable and were not compromised by different types of exercise training. Serum testosterone and testosterone/cortisol ratio increased over the 19‐week study period, while cortisol levels remained unchanged in both groups, suggesting an improved anabolic hormonal milieu. These findings are in line with Santtila et al. (2009), who reported an increase of 17%–27% in testosterone and an increase (or no change) in testosterone/cortisol ratio in Finnish male conscripts during an 8‐week basic training period, regardless of training interventions focusing on endurance or strength. Similarly, Ojanen et al. (2020) reported increased testosterone/cortisol ratio during 12 weeks of military service, despite different physical training modalities between the study groups. In contrast to the present data, a decline in testosterone and an increase in cortisol during military training have been demonstrated in several studies. However, these changes are typically observed during short‐term field training periods (Kyröläinen et al., 2008) or a more intensive military training (Nindl et al., 2007). Despite the positive changes in testosterone and testosterone/cortisol ratio in the present study, it can be discussed whether this was a true response to the military training environment or could it be more of a rebound to normal levels prior to military service. Baseline testosterone levels were at the lower end of the normal reference range (10–38 nmol·L^−1^) in both groups. This raises a question of whether these levels had already decreased due to the drastic transition from civilian life to the military environment with multiple stressors (Beckner et al., 2022). A comprehensive understanding of hormonal response patterns during military training requires baseline measurements to ideally be conducted prior to the initiation of service. However, such measurements are often difficult to implement due to practical constraints.

IGF‐1 is considered as a sensitive biomarker, which typically decreases substantially during periods of heavy physical stress in soldiers (Gomez‐Merino et al., 2004; Nindl et al., 2007). However, there are conflicting findings on responses in circulating IGF‐1 during both acute and chronic exercise training (Frystyk, 2010). An increase in IGF‐1 between PRE and POST was observed only in CON. While IGF‐1 was elevated in EXP in MID, the observed increase was diminished in POST. This difference in IGF‐1 response could be due to the fact that the EXP group underwent a 5‐day live‐fire exercise in the week before POST measurements, which may have caused a decline in IGF‐1 due to physical stress and energy deficit (Nindl & Pierce, 2010). Previous studies have shown that the recovery of IGF‐1 levels after strenuous military training can take at least 1 week (Henning et al., 2014; Vikmoen et al., 2020). Another relevant consideration regarding IGF‐1 is the role of changes in body composition. Changes in body fat mass and body mass were weakly and inversely correlated with the change in IGF‐1 in EXP, which has also been observed in other military studies (Nindl et al., 2007, 2012). However, distinguishing the specific contribution of alterations in body composition is challenging, as the regulation of IGF‐1 is a complex interplay between body composition, and nutritional and training status. While the exact mechanisms are yet to be established, it seems that exercise training or increased physical activity and increased energy flux can cause a decrease in circulating IGF‐1, also in the absence of negative energy balance or weight loss (Nemet et al., 2004; Rarick et al., 2007). This controversy between the known anabolic role and the possible decrease in circulating IGF‐1 during exercise training further complicates the interpretation of different responses.

Previous studies have reported increases (Drain et al., 2017; Tanskanen et al., 2011) or no change (Santtila et al., 2009) in SHBG during military training. These findings are consistent with the present results, as SHBG increased in EXP while no change occurred in CON. However, it remains unclear whether the observed increase in SHBG in EXP was specifically caused by the exercise intervention or by other factors related to military training. In a similar training intervention study by Drain et al. (2017), an increase in SHBG was observed in a high‐intensity training group and a control group. Furthermore, Ojanen et al. (2020) reported an increase in SHBG in Finnish conscripts over a 12‐week strength training or anaerobic training period, but no change occurred in the control group, which trained with a similar program as the control group in the present study. While high overall physical activity is associated with higher SHBG concentrations, this association is largely attenuated after adjustment for body mass index (Watts et al., 2021). The increase in SHBG in EXP may partly be explained by the observed relationship between the decrease in body mass, as SHBG is inversely associated with body mass index (Watts et al., 2017), and increases in SHBG have also been reported in weight loss studies (Niskanen et al., 2004; Telgenkamp et al., 2019). Similar to the present study, an inverse association between changes in body mass/body fat and change in SHBG has also been observed in soldiers (Drain et al., 2017; Henning et al., 2014). In general, SHBG appears to be influenced not only by changes in body composition but also by alterations in metabolic processes, such as hepatic de novo lipogenesis, which has been shown to be inversely associated with SHBG levels (Simons et al., 2021). Low SHBG levels are also associated with several human diseases and are predictive of cardiometabolic risks, such as type 2 diabetes and cardiovascular disease (Simó et al., 2015). While such associations in the present study remain speculative, the observed increase in SHBG accompanied by a decrease in body mass in EXP may reflect favorable changes in health parameters, similar to the observed changes in inflammatory biomarkers in the current study.

Although adverse responses in inflammatory biomarkers in soldiers have been documented in several studies, these findings are often made in the context of more physically strenuous military training (Beckner et al., 2022). In the present study, hs‐CRP and IL‐6 decreased in both groups, with values remaining at levels expected for healthy individuals across all measurement points. TNF‐α remained unaltered in CON, and while the ~24% relative increase in EXP was statistically significant, the absolute change from 0.8 ± 0.2 to 1.0 ± 0.2 pg mL^−1^ is unlikely to hold clinical relevance, as these values are well within the normal reference limit of <8.1 pg mL^−1^. These positive changes in both groups align with existing literature on the impact of regular military training on inflammatory responses. Tait et al. (2021) reported only minimal changes in inflammatory biomarkers during the 12‐week Australian Army basic military training and concluded that the training loads were adequate to support recruit readiness and well‐being. Similarly, during a 4‐month Israeli Army basic combat training, Nindl et al. (2012) observed decreases in TNF‐α and no change in IL‐6, accompanied by an increase in IGF‐1, indicating a favorable physiological state. It appears that well‐structured military training does not induce detrimental effects on inflammatory biomarkers but rather promotes the development of a balanced inflammatory status.

The absence of notable between‐group differences in inflammatory biomarkers limits the interpretation of the independent effect of the current training intervention. We did not expect HIFT to be more effective than conventional training in reducing inflammation, as there is no conclusive evidence suggesting that inflammation responses are directly determined by training intensity. Exercise training of any intensity can reduce inflammation, also in the absence of weight loss (Fedewa et al., 2017; Rose et al., 2021). However, higher CRP is strongly associated with obesity (Choi et al., 2013), highlighting the relevance of alterations in body composition. In the present study, changes in hs‐CRP were weakly to moderately associated with changes in body mass and fat mass in both groups, suggesting that reductions in hs‐CRP may be more strongly influenced by improvements in body composition than by training modality. The favorable downward trend in inflammatory biomarkers may be attributed to the combined effects of the alterations in body composition and the overall physical activity during military training. In line with this, the associations between changes in physical fitness and the measured biomarkers were modest, suggesting that the training‐induced biomarker responses did not strongly explain the observed improvements in fitness.

A major strength of this study is that it was conducted in an authentic military training environment, which enhances the practical applicability of the findings. The relatively long intervention period further strengthens the reliability of the observed adaptations, and the inclusion of both physical performance tests and biomarker analyses provided a multifaceted view of training responses. Some limitations must also be acknowledged. The findings cannot be directly generalized to female soldiers, older age groups, or soldiers in different military contexts. Biomarker outcomes are subject to natural inter‐individual variability, which may affect the detection of small changes. Furthermore, we were unable to control or systematically monitor participants' leisure‐time physical activity, the overall training load from other military duties, or lifestyle factors such as diet, sleep, and stress, all of which could have influenced the results. Groups were assigned by company rather than individual randomization, which may introduce unit‐level confounding. Finally, the exact training intensity and external load of the intervention were not objectively tracked throughout the study.

## 5 CONCLUSION

The present results suggest that HIFT had no negative impact on serum hormones or inflammatory biomarkers during military service when compared to traditional physical training, so it can be inferred that the total physiological strain was not excessive. As part of the current work, we recently reported that HIFT is an effective and time‐efficient approach to improve physical performance in soldiers (Helén et al., 2023). The findings from the present study support the compatibility of HIFT with other demands of military training, as it also appears to be well tolerated with respect to hormonal and inflammatory responses within the overall training load of military service. It is important to note that these results may not be universally applicable to military units with more rigorous training requirements. In pursuit of optimal physical training strategies in military populations, the total physiological strain must always be considered and objectively monitored or evaluated by subject matter experts. In conclusion, HIFT does not appear to induce negative responses in serum hormones or inflammatory biomarkers and can be recommended for integration into military training when total training load is also monitored.

## FUNDING INFORMATION

This study was funded by the Finnish Defence Forces Defence Command and the National Defence Foundation.

## CONFLICT OF INTEREST STATEMENT

No conflict of interest declared.

## ETHICS STATEMENT

This study was approved by the Ethical Committee of the Central Finland Health Care District (HUS‐1557‐2018‐8) and the Finnish Defence Forces (AP10027). All participants gave written informed consent prior to participation in the study, and the procedures complied with the Declaration of Helsinki.

## Data Availability

The datasets generated and/or analyzed during the current study are available from the corresponding author upon reasonable request.
